# Postoperative Vision Loss in Shoulder Surgery: A Case Series and Review of the Literature

**DOI:** 10.7759/cureus.97261

**Published:** 2025-11-19

**Authors:** J. Anthony Chacko, Reece Mitchell, J. Ryan Hill, Samantha A Mohler, Jennifer I Doyle, Joseph G Chacko, Sami Uwaydat

**Affiliations:** 1 Department of Ophthalmology, University of Arkansas for Medical Sciences, Little Rock, USA; 2 Department of Orthopaedic Surgery, University of Arkansas for Medical Sciences, Little Rock, USA; 3 Department of Ophthalmology, Little Rock Eye Clinic, Little Rock, USA

**Keywords:** electroretinography, erg, ion, ischemic optic neuropathy, postoperative vision loss, povl, shoulder arthroplasty/replacement, shoulder surgery

## Abstract

Postoperative vision loss (POVL) can be devastating and permanent. We present three unique cases of POVL after shoulder surgery, and we provide recommendations on patient positioning and eye protection perioperatively based on our experience and a review of the literature.

In Case 1, our patient underwent an uncomplicated right arthroscopic rotator cuff repair and acromioclavicular (AC) joint resection in the beach-chair (BC) position. He developed bilateral vision loss (right eye greater than the left eye) in the postoperative period, with visual field deficits noted on Humphrey visual field (HVF) 24-2, and decreased retinal function on electroretinography (ERG) in the right eye (OD). In Case 2, our patient underwent an uncomplicated left total shoulder arthroplasty with open biceps tenodesis and implant removal in the BC position. He developed vision loss in the left eye (OS) in the postoperative period, with visual field deficits noted on HVF 24-2, and ERG findings that pointed to an ischemic injury to the retina and optic nerve in the left eye (OS). In Case 3, our patient underwent an uncomplicated right arthroscopic rotator cuff repair in the BC position. He developed vision loss OS in the postoperative period and was diagnosed with a macular-sparing central retinal artery occlusion (CRAO) OS, with eventual foveal recovery. POVL after shoulder surgery is a serious condition with a risk of permanent vision loss. Although POVL after shoulder surgery is rare and likely multifactorial, it is important to minimize risks and optimize both patient positioning and eye protection to promote patient safety and favorable outcomes.

## Introduction

Postoperative vision loss (POVL) is a rare but serious complication that can occur in non-ocular surgery. The incidence of POVL ranges from 0.0009% to 16.3%, with prone-position spine surgery and cardiopulmonary bypass procedures having the highest incidence [1]. POVL onset varies from immediately upon awakening to 10 days postoperatively, with the median onset occurring at 15 hours [2]. POVL can present unilaterally or bilaterally as decreased vision, ophthalmoplegia, or ptosis [3]. POVL progression to postoperative blindness occurs in 0.1%-1.0% of cases [1]. While the pathophysiology of POVL is not completely understood, hypoperfusion to essential neural tissues along the visual pathway is a common mechanism [1].

The prognosis of POVL is poor, with permanent visual loss occurring in most cases [2]. Risk factors for POVL include preoperative anemia, intraoperative blood loss >1000 mL, procedures longer than six hours, hypotension, increased intraocular pressure (IOP), and systemic vascular diseases such as atherosclerosis, hypertension, and diabetes [2,4,5]. Ischemic optic neuropathy is the most cited etiology of POVL resulting from orthopedic surgery [1]. We present three unique cases of POVL following shoulder surgery.

## Case presentation

Case 1

A 58-year-old Caucasian male with a past medical history (PMH) of migraine headache with aura and hypothyroidism presented with vision loss in the right eye following right arthroscopic rotator cuff repair and acromioclavicular (AC) joint resection the previous day.

The procedure was performed without complication in the beach-chair (BC) position with a face shield. Minimal blood loss was reported, and he remained hemodynamically stable. Upon awakening, he reported decreased vision in the right eye (OD) greater than the left eye (OS), with “dark shadows” in his vision bilaterally. On physical examination, an anesthesiologist determined his visual acuity (VA) to be at least hand motion OD and count fingers (CF) at 2 feet OS. Confrontational visual fields (CVFs) were intact in all four quadrants OS. A magnetic resonance imaging (MRI) study of the brain, with and without contrast, revealed no cerebral infarction.

**Figure 1:**
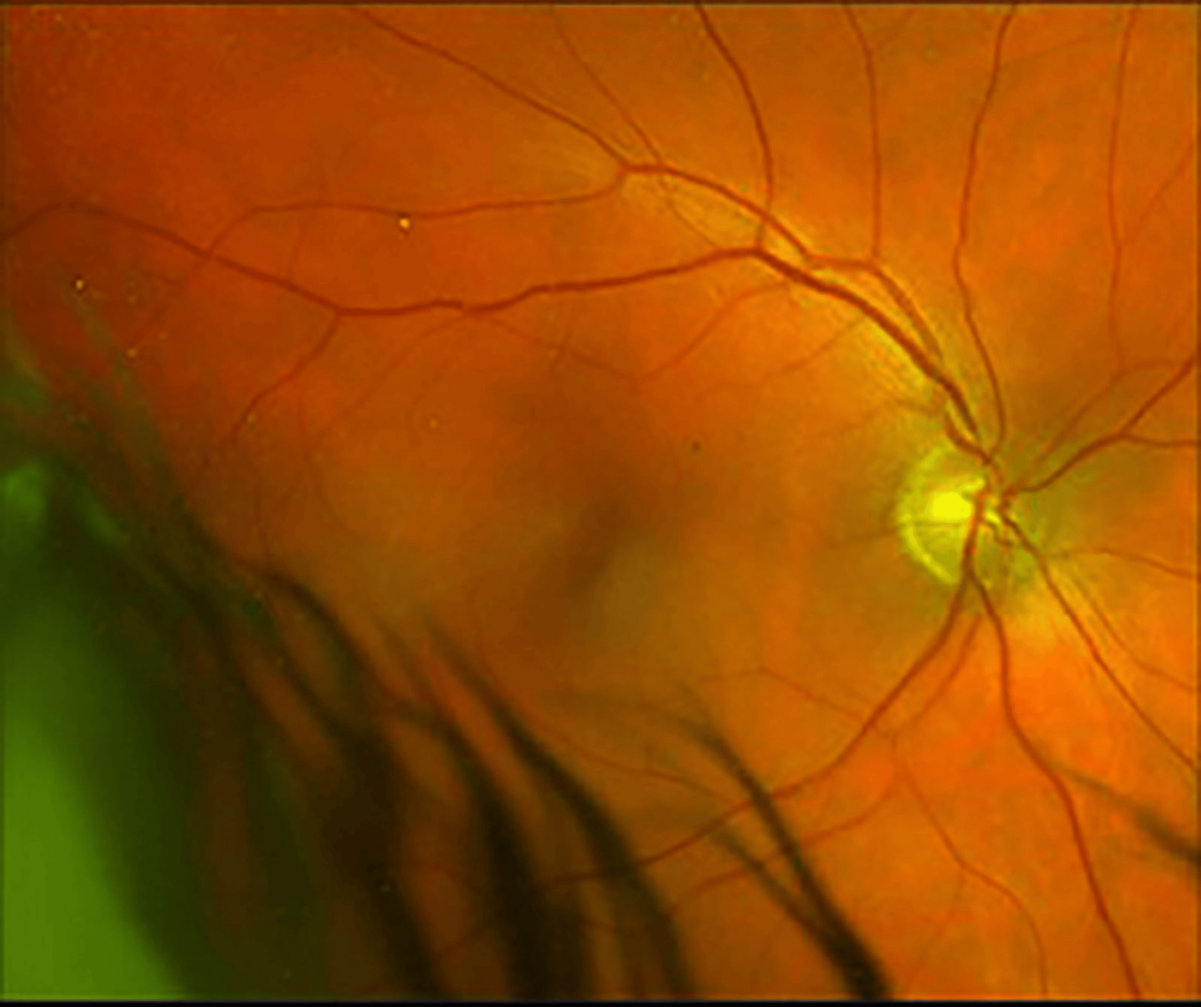
Fundus photography OD - normal fundus OD OD, right eye

**Figure 2:**
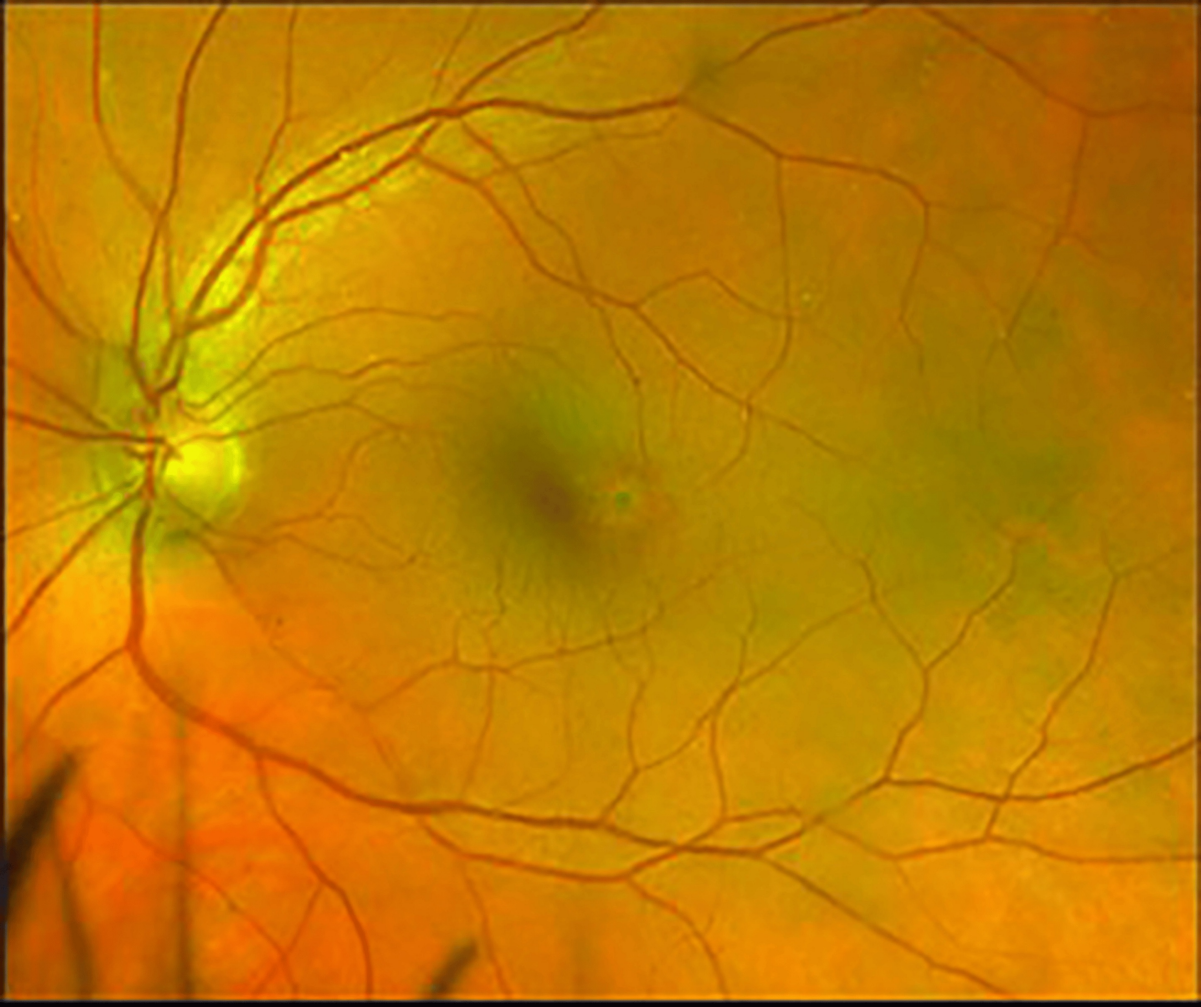
Fundus photography OS - stable temporal macular scar OS OS, left eye

**Figure 3:**
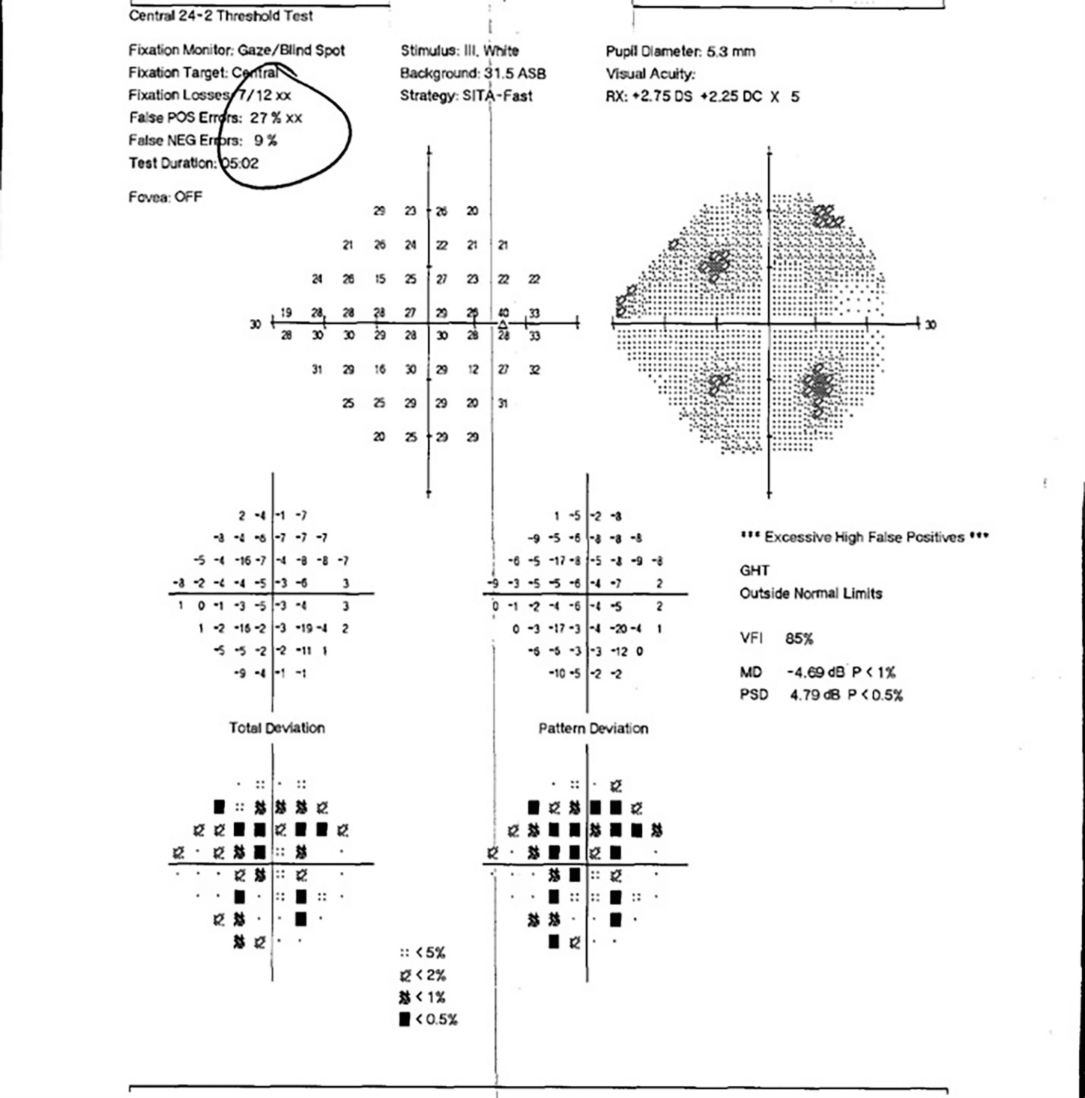
Humphrey visual field (HVF) 24-2 OD - unreliable, with generalized depression OD OD, right eye

**Figure 4:**
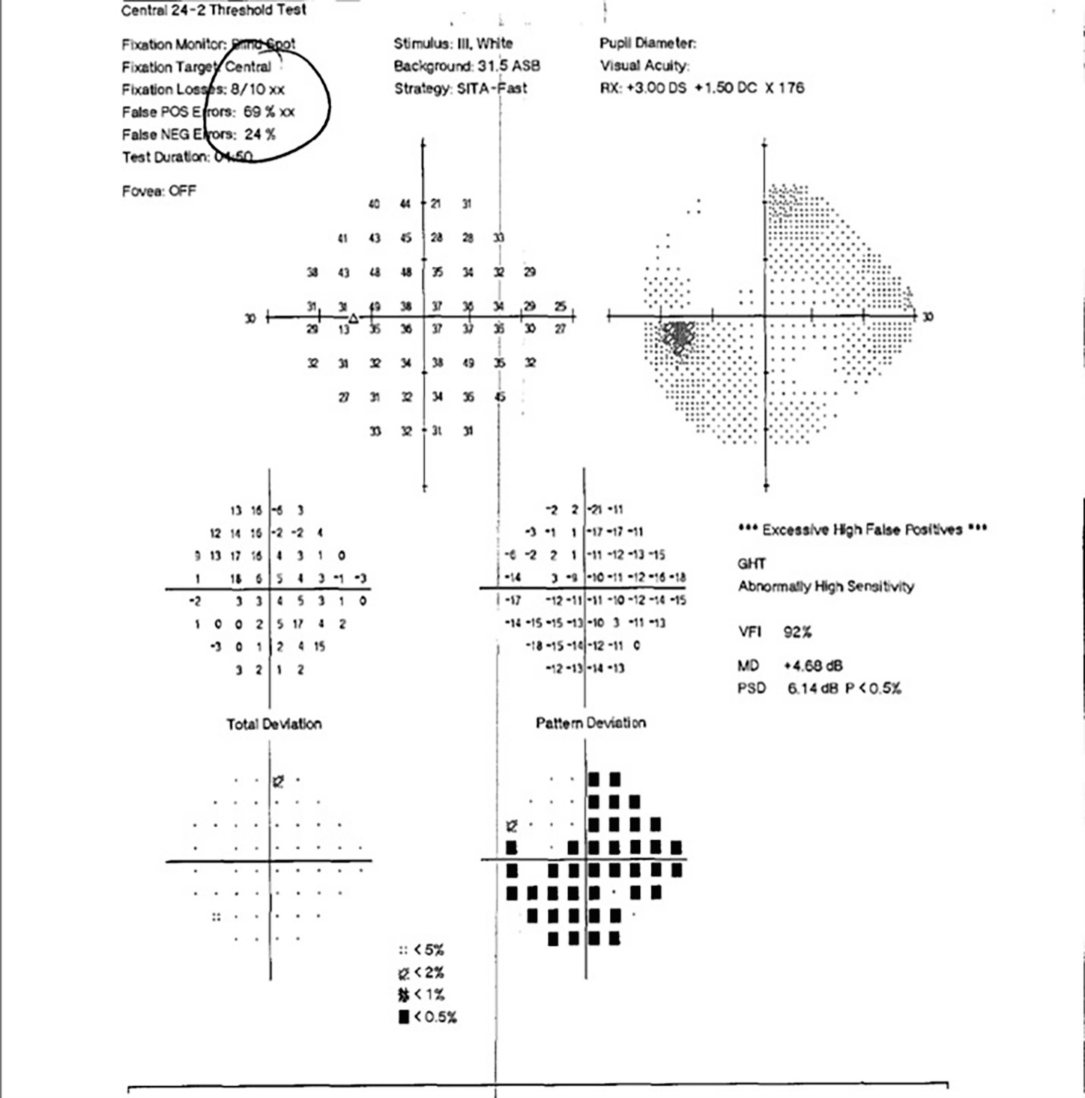
Humphrey visual field (HVF) 24-2 OS - unreliable, with nonspecific defects OS OS, left eye

In the neuro-ophthalmology clinic the next day, the patient stated he “could not see at all” after waking up from surgery. He denied eye pain, headache, scalp tenderness, or jaw claudication. Ophthalmologic examination revealed a corrected VA of 20/20 OU (both eyes). IOP and CVF were normal OU. A dilated fundus exam (DFE) was within normal limits (WNLs) OU, with pink and sharp optic discs and a focal macular scar noted OS (Figures 1-2). Humphrey visual field (HVF) 24-2 showed a generalized depression OD, worse superiorly (Figures 3-4). The left field was unreliable.

**Figure 5:**
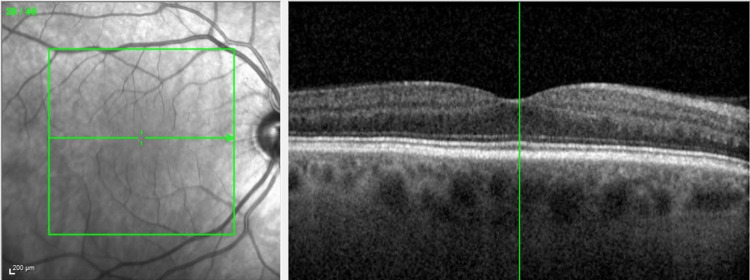
OCT macula OD - unremarkable OD OCT, optical coherence tomography; OD, right eye

**Figure 6:**
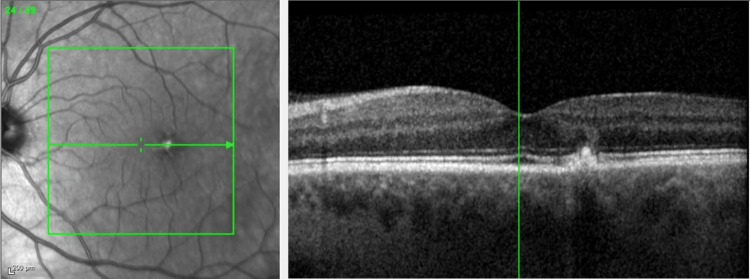
OCT macula OS - stable temporal macular scar OS OCT, optical coherence tomography; OS, left eye

He was referred to the retina clinic two months later for persistent visual disturbances and nyctalopia OD. Ocular examination was stable. Optical coherence tomography (OCT) was unremarkable OU, with a stable focal macular scar noted OS (Figures 5-6). On full-field electroretinography (FF ERG), the rod and cone amplitudes were reduced OD greater than OS, which was consistent with decreased retinal function OD.

Case 2

A 62-year-old Caucasian male with a PMH of obstructive sleep apnea on continuous positive airway pressure (CPAP), dyslipidemia, hypertension, coronary artery disease status post stenting, and mild mitral regurgitation experienced vision loss OS following left total shoulder arthroplasty with open biceps tenodesis and implant removal. The procedure was performed in the BC position without complication or hemodynamic instability. The patient had decreased vision and subjective eye pressure OS immediately in the postoperative period. MRI of the brain and orbits, with and without contrast, revealed no acute intracranial abnormality, nor any acute abnormalities in the orbits or optic nerves.

**Figure 7:**
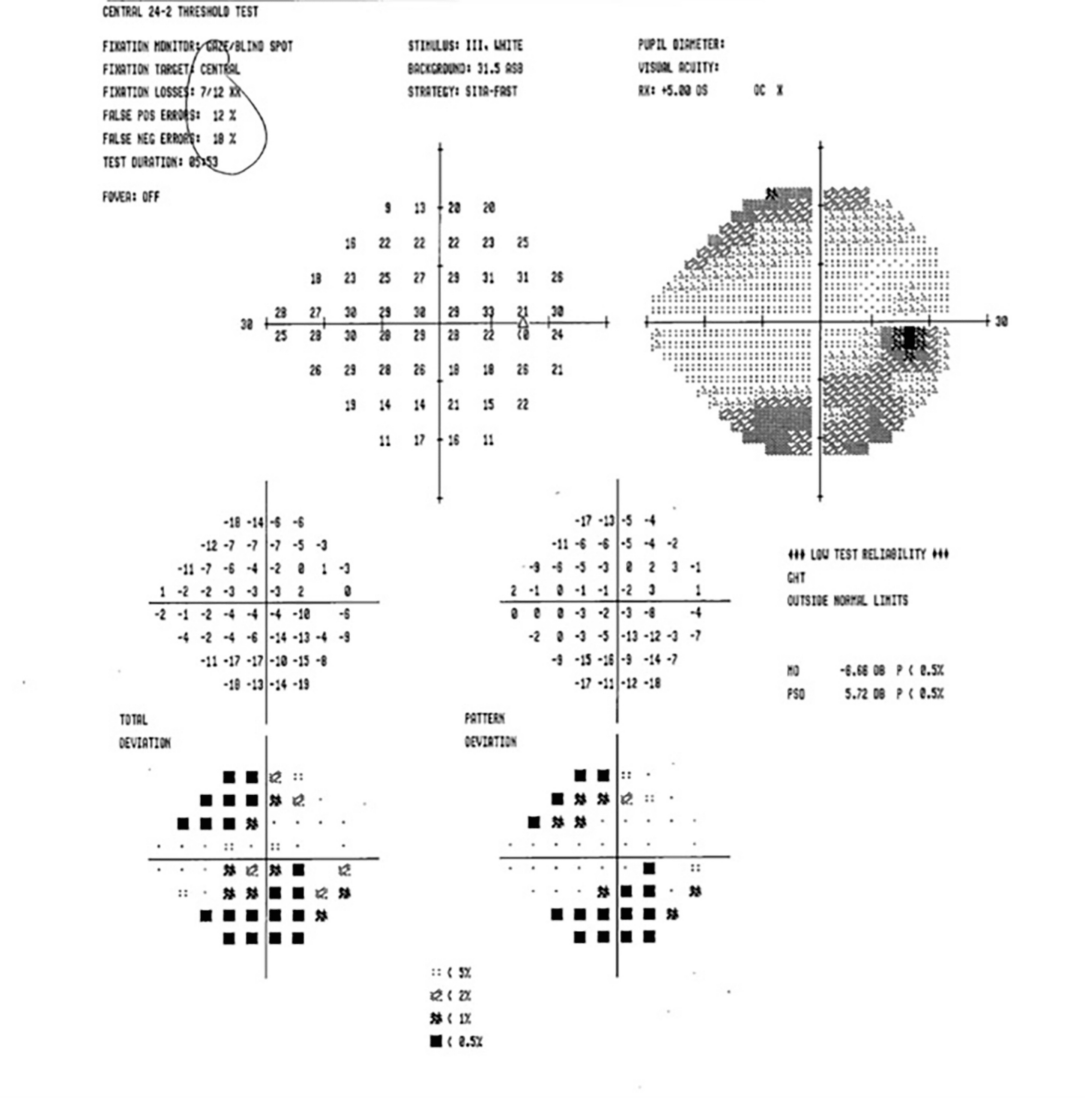
HVF 24-2 OD - unreliable, with nonspecific inferior arcuate and superonasal defects OD HVF, Humphrey visual field; OD, right eye

**Figure 8:**
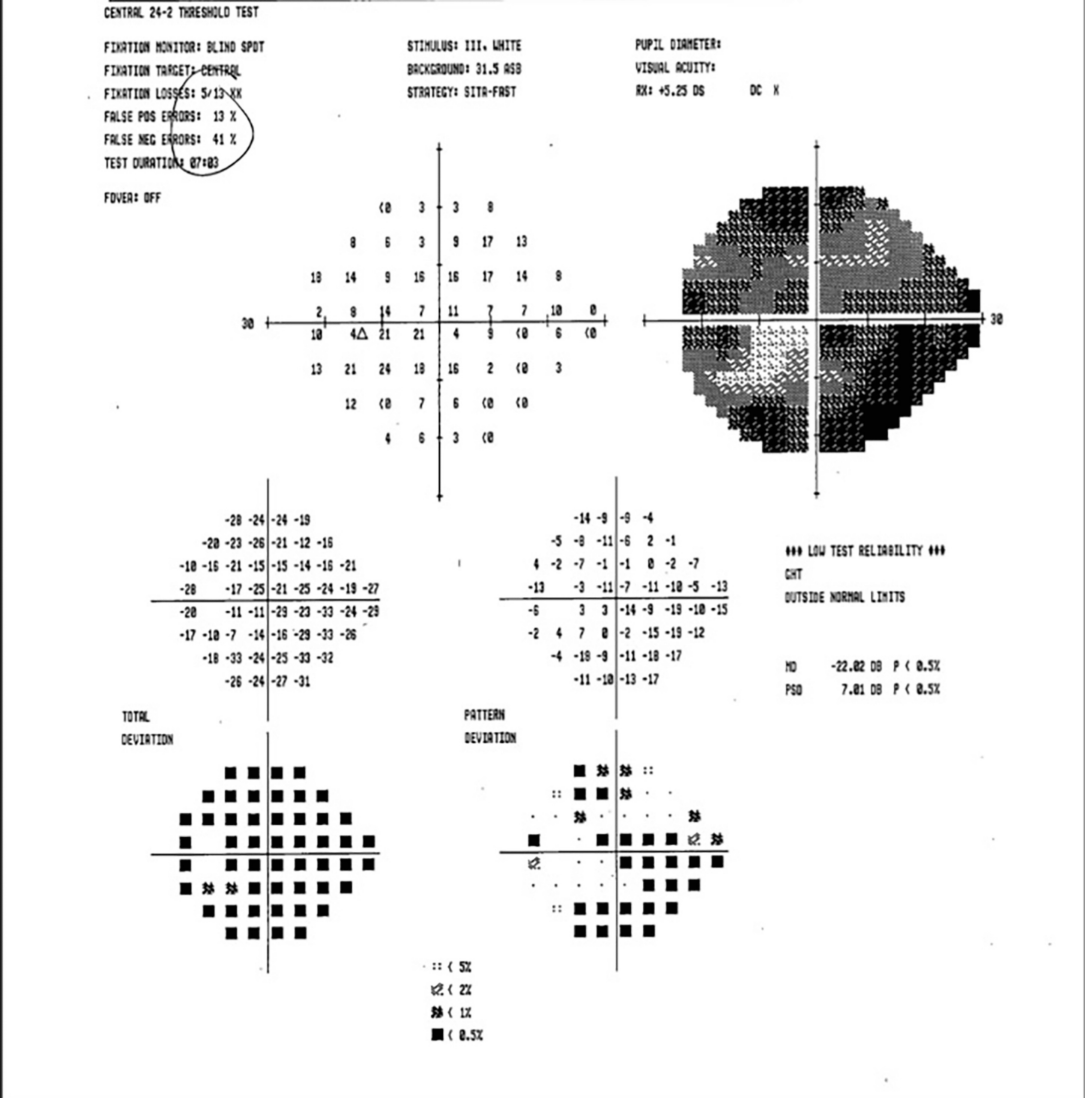
HVF 24-2 OS - unreliable, with generalized depression and constriction OS HVF, Humphrey visual field; OS, left eye

In the neuro-ophthalmology clinic two weeks later, an examination revealed a corrected VA of 20/20 OD and 20/25 OS. IOP and CVF were normal OU. There was a relative afferent pupillary defect (RAPD) OS. DFE revealed pink and sharp optic discs, with a few dot-blot hemorrhages noted OD. HVF 24-2 showed nonspecific inferior and superonasal defects OD; in the left eye, the test was unreliable (Figures 7-8).

**Figure 9:**
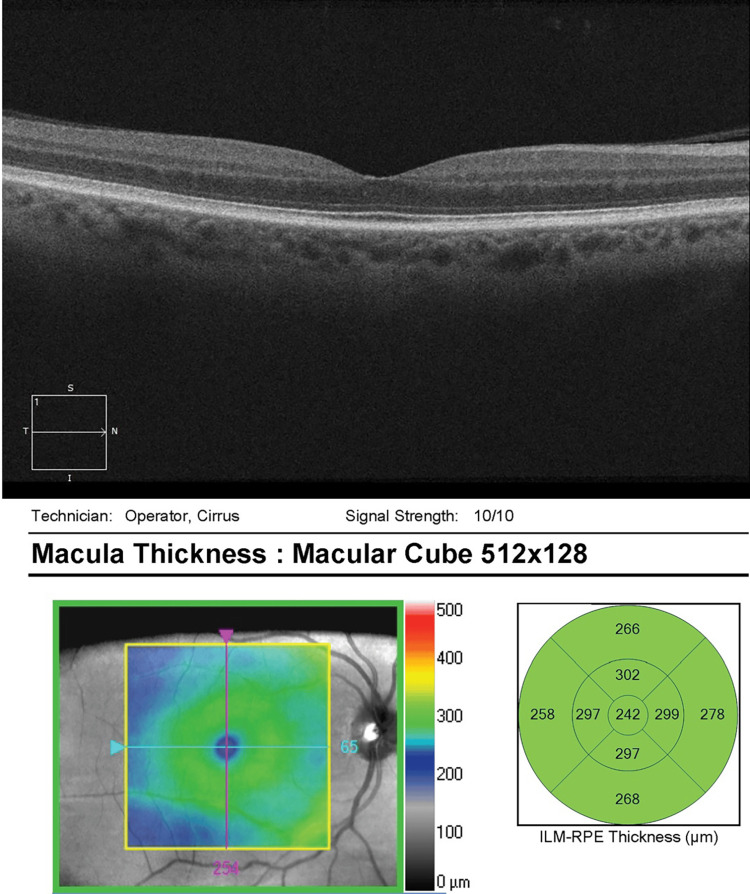
OCT macula OD - normal OCT OD OCT, optical coherence tomography; OD, right eye

**Figure 10:**
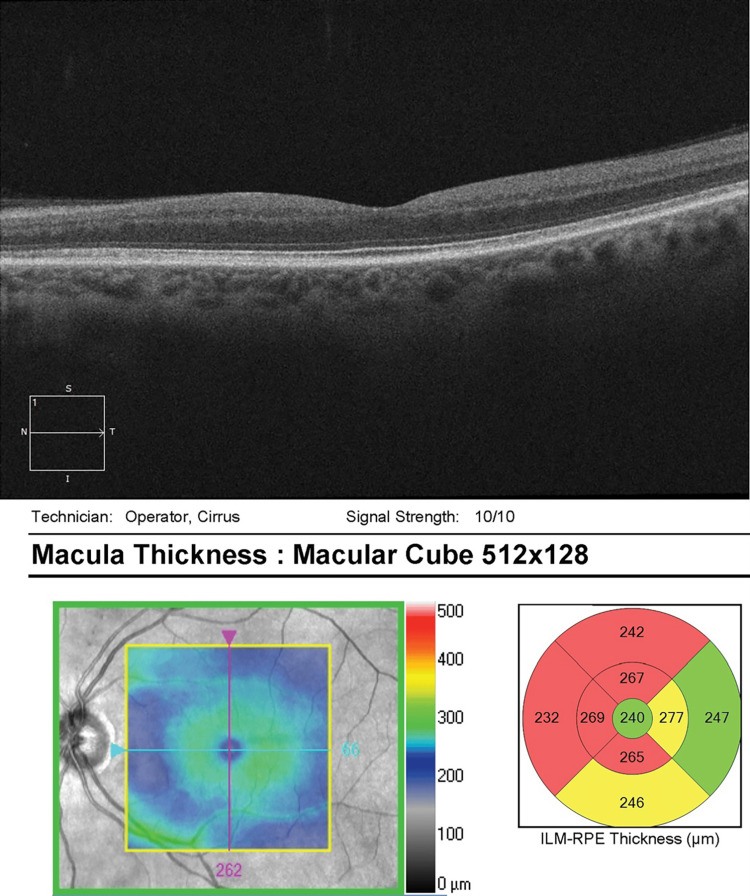
OCT macula OS - superonasal macular thinning OS OCT, optical coherence tomography; OS, left eye

He was referred to the retina clinic two months later for persistent visual loss OS. Examination showed corrected VA improvement to 20/20 in the left eye. OCT macula was normal OD, but documented superonasal macular thinning OS (Figures 9-10). On FF ERG, the amplitudes and implicit times were reduced OS greater than OD, consistent with an ischemic injury to the retina.

Case 3

A 57-year-old Black male with primary open-angle glaucoma OU and a PMH of type II diabetes mellitus and hypertension underwent a right arthroscopic rotator cuff repair with subacromial decompression and shoulder joint debridement. The procedure was performed in the BC position without complications. The patient remained hemodynamically stable, with protective eye goggles in place throughout the case, and experienced minimal blood loss. In the immediate postoperative period, he experienced sudden-onset, painless vision loss OS.

In the emergency department, he denied any eye pain, headache, scalp tenderness, or jaw claudication. Examination revealed a VA of 20/25 OD and CF at 1 foot OS. CVFs were full OD. An RAPD was noted OS. Examination of the unaffected right eye was normal. DFE OS revealed retinal edema with a cherry-red spot, consistent with a central retinal artery occlusion (CRAO) OS. No embolus was identified. He was admitted for a comprehensive stroke work-up. Erythrocyte sedimentation rate (ESR) and C-reactive protein (CRP) were elevated at 34 mm/hr and 68.8 mg/L, respectively. In the absence of giant cell arteritis (GCA) symptoms, the elevated inflammatory markers were likely due to postoperative inflammation. MRI of the brain and computed tomography angiography (CTA) of the head and neck revealed no acute intracranial findings or hemodynamically significant stenosis. The transthoracic echocardiogram was unremarkable. OCT macula showed hyperreflectivity and a lack of stratification of the inner retinal layers OS. Anterior chamber paracentesis was performed OS. He was started on aspirin 81 mg and atorvastatin 40 mg daily, and discharged the next day.

**Figure 11:**
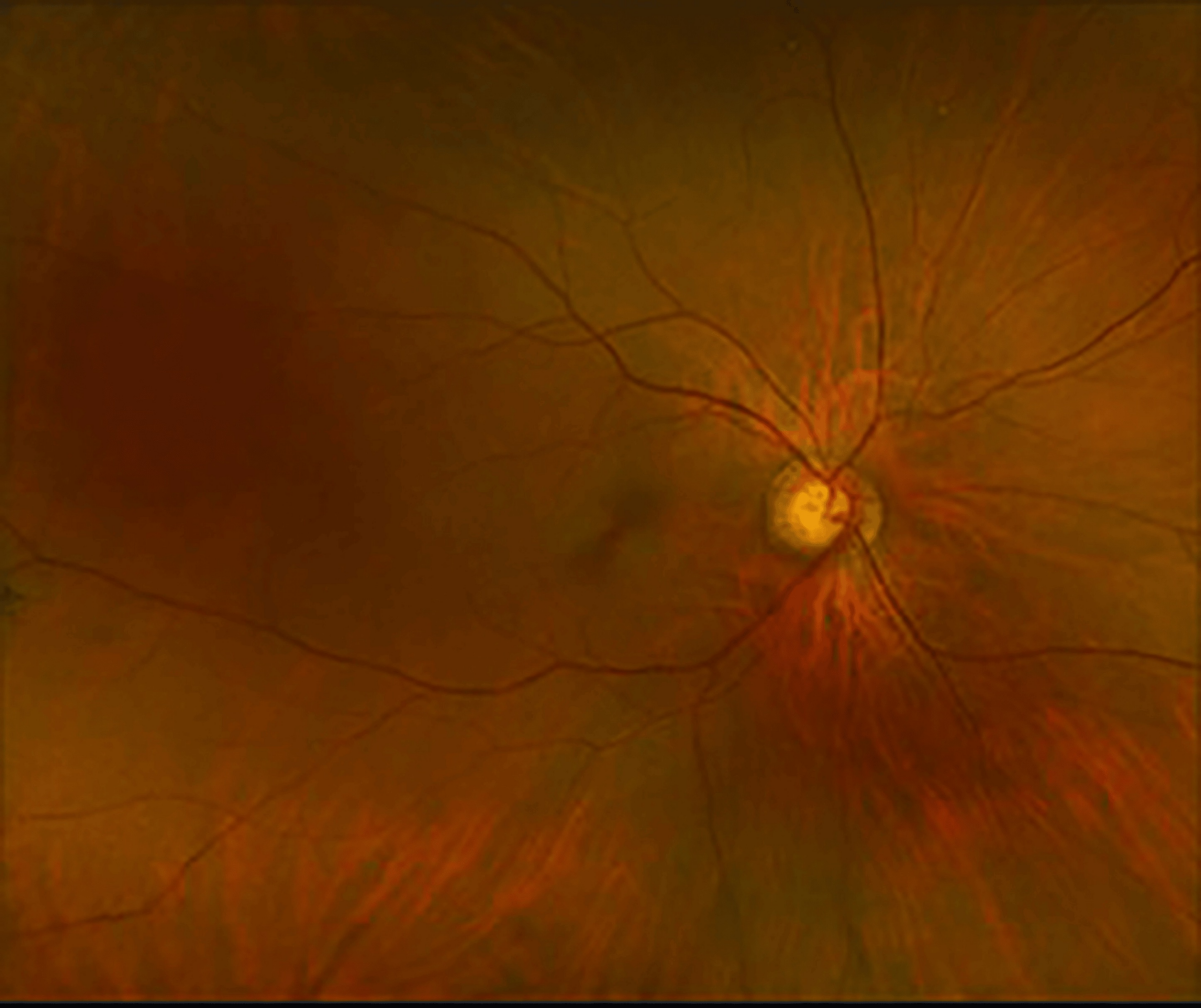
Fundus photography OD - normal fundus with glaucomatous cupping OD OD, right eye

**Figure 12:**
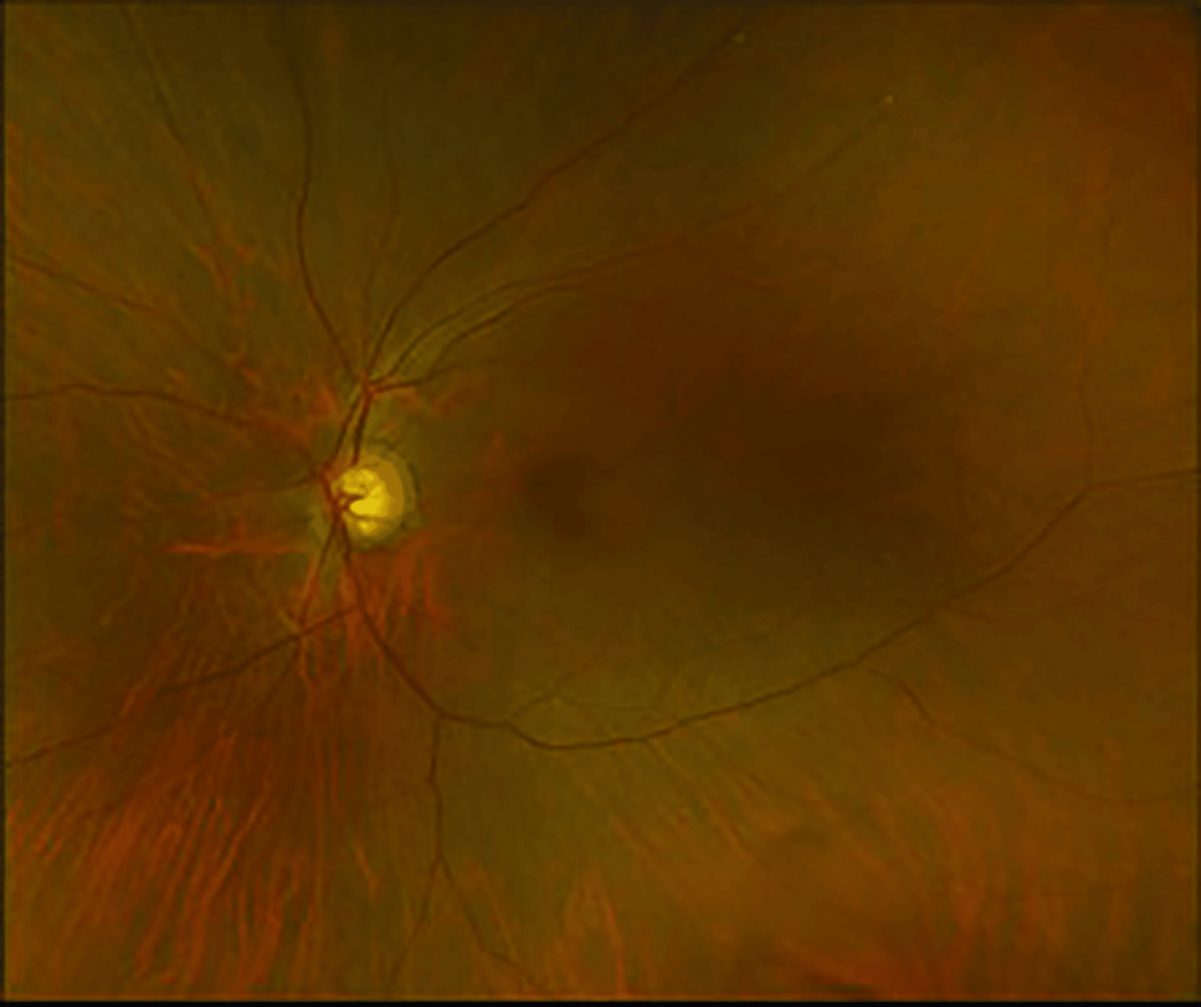
Fundus photography OS - glaucomatous cupping with macular atrophy and vascular attenuation OS OS, left eye

**Figure 13:**
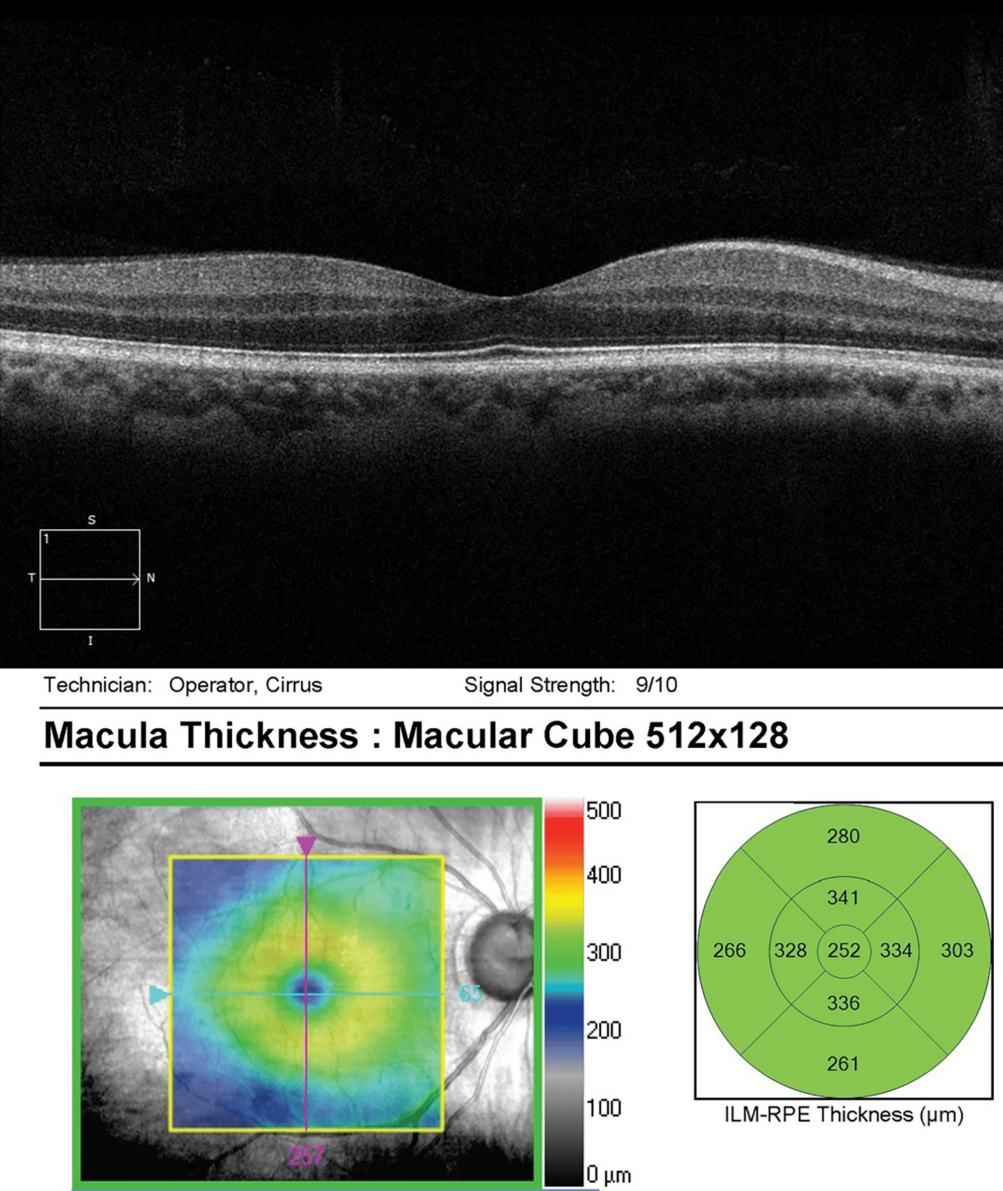
OCT macula OD - normal OCT OD OCT, optical coherence tomography; OD, right eye

**Figure 14:**
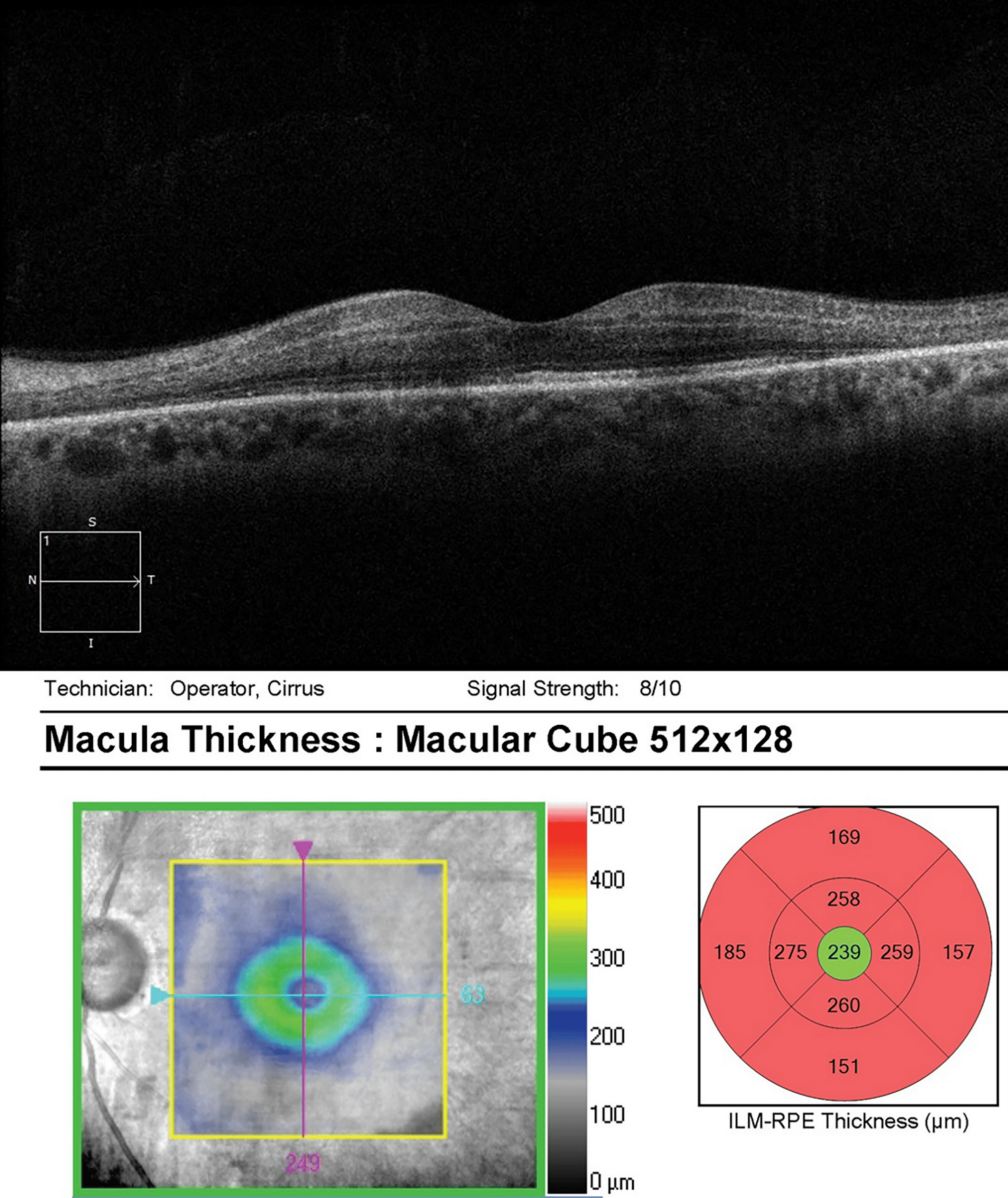
OCT macula OS - macular atrophy with foveal sparing OS OCT, optical coherence tomography; OS, left eye

**Figure 15:**
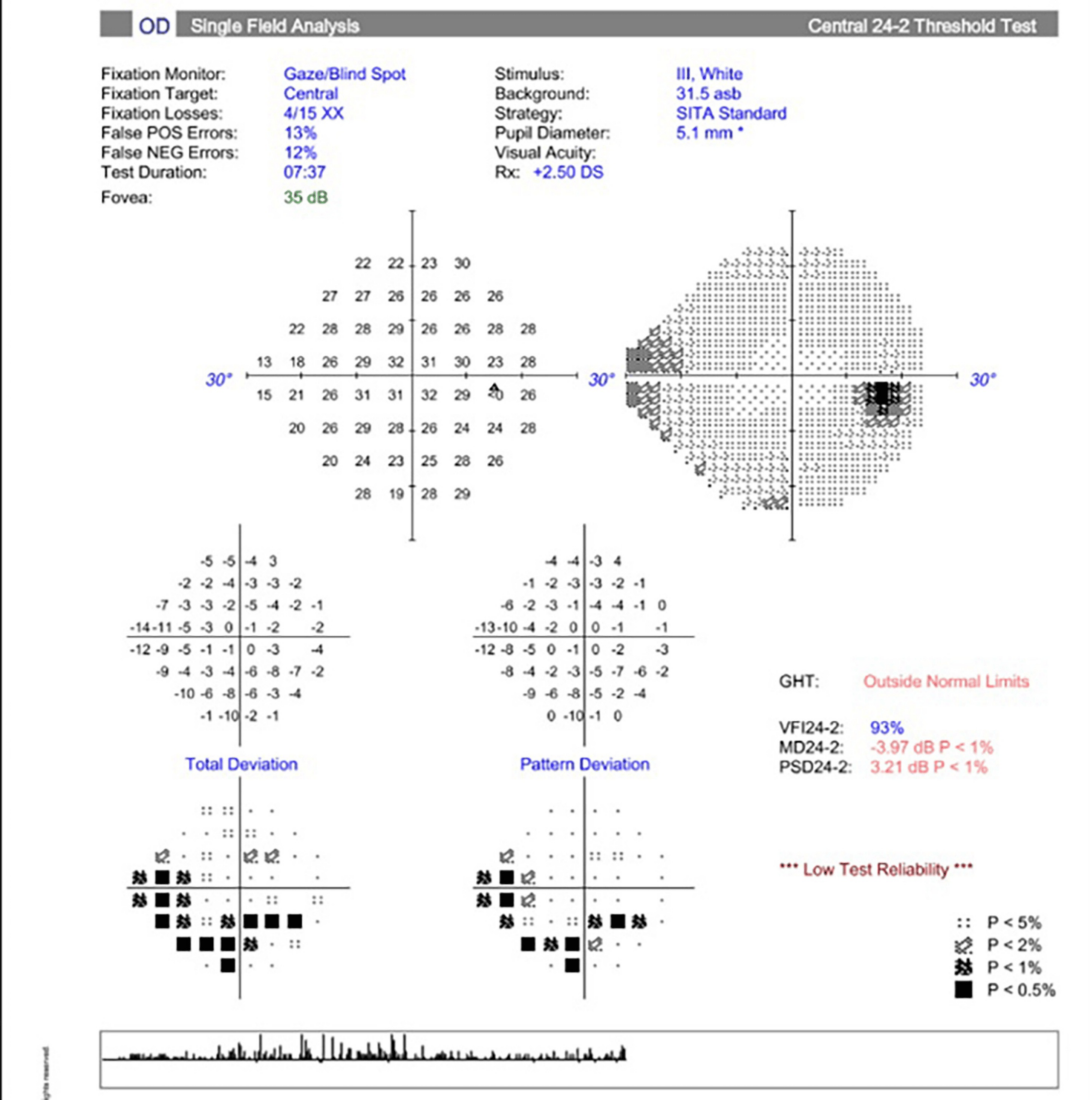
HVF 24-2 OD - unreliable with nasal defect OD likely secondary to glaucoma HVF, Humphrey visual field; OD, right eye

**Figure 16:**
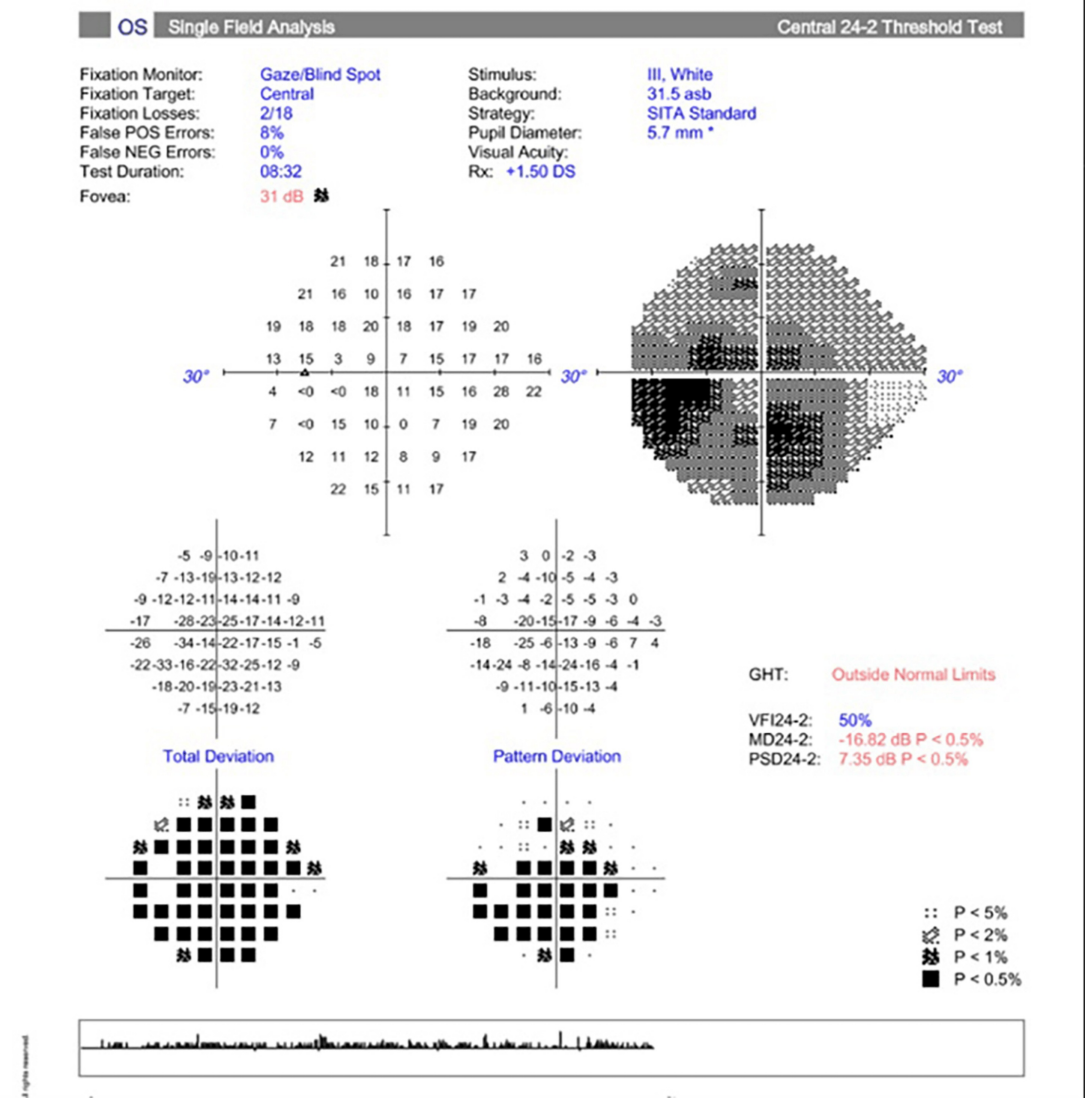
HVF 24-2 OS - reliable with central scotoma OS HVF, Humphrey visual field; OS, left eye

At the three-week follow-up, the patient continued to have visual field loss OS. Best-corrected VA improved to 20/20 OS. There was macular atrophy and vascular attenuation OS (Figures 11-12). OCT macula revealed inner retinal atrophy OS, with foveal sparing (Figures 13-14). HVF 24-2 OS showed a central scotoma (Figures 15-16). He was diagnosed with a CRAO OS, with foveal recovery.

## Discussion

POVL in shoulder surgery is a rare complication. While the incidence of POVL in shoulder surgery is unknown, there have been five reported cases of POVL associated with shoulder surgery [1,4-7]. In these cases, the etiologies of POVL include unilateral vision loss and external ophthalmoplegia [1], anterior ischemic optic neuropathy (AION) [4], posterior ischemic optic neuropathy (PION) [5], AION due to ocular compression [6], and CRAO [7]. In our cases, varying etiologies of POVL are represented, specifically mixed retinal and optic nerve ischemia and CRAO.

The etiology of POVL in shoulder surgery is likely multifactorial, with surgical and anesthetic complications, and patient risk factors playing a significant role. Risk factors for POVL include male sex, obesity, preoperative anemia, and peripheral vascular disease [1]. Janarek and Colechá explain that defective vascular autoregulation in a patient with atherosclerotic disease can increase the susceptibility of the intraorbital optic nerve to ischemic events [5]. Although the pathophysiology of POVL is not fully understood, Su et al. state that the common underlying mechanism appears to be insufficient vascular perfusion to the visual pathway [8]. Blood flow to the optic nerve may be compromised by decreased mean arterial pressure (MAP), increased IOP, or increased arterial resistance [9].

In shoulder surgery, Li et al. state that deliberate hypotensive anesthesia is an effective technique for reducing blood loss and maintaining a clear surgical field [10]. The current expert recommendation is to keep the systolic blood pressure (SBP) >90 mmHg, and the maximum reduction of both the SBP and MAP <20% of baseline measurements to prevent cerebral hypoperfusion [10]. However, intraoperative hypotension can result in POVL. In two reports, intraoperative prolonged hypotension combined with vascular risk factors during shoulder surgery (MAP reduced by almost 51% to 51 mmHg for 95 minutes [5], and MAP reduced by 41.6% for 80 minutes [4]) resulted in unilateral PION and unilateral AION in two patients, respectively. Gilbert et al. explain that the perfusion pressure of the eye drops linearly with the mean arterial blood pressure [4]. Hypoperfusion of the optic nerve is the most important factor in developing PION [5]. In our cases, each patient experienced minimal blood loss and remained hemodynamically stable, with SBP >90 mmHg. In the absence of significantly decreased blood volume or MAP, we hypothesize that intraoperative hypoperfusion to the optic nerve is likely derived from external forces, leading to increased IOP via patient positioning or the use of a face shield.

Shoulder surgery can be performed using the lateral decubitus (LD) position or the BC position [10,11]. The advantages of the LD position are better visualization and instrument access, but the disadvantages include difficulties in airway management and/or anesthetic delivery [11]. In the BC position, the patient’s trunk and head are elevated 30° to 90°, with the head secured in a headrest using well-padded straps. Advantages of the BC position include ease of setup, anatomical orientation, and ready conversion from arthroscopic to open approach if required [10,11]. Disadvantages of the BC position include the risk of cerebral hypoperfusion, blindness, cranial nerve injury, cervical traction neurapraxia, and cardiac and embolic events [10]. Murphy et al. report a substantially higher incidence of cerebral desaturation events (CDEs) during shoulder arthroscopy in patients positioned in the BC position versus the LD position (80.3% versus 0%; CDEs were defined as a ≥20% decrease in baseline regional cerebral oxygen saturation) [12]. In the BC position, Li et al. state that “the head must be in a neutral position at all times because extension and rotation can reduce vertebral artery blood flow, resulting in posterior brain circulation infarcts, and flexion can impede cerebral venous drainage by causing obstruction of the internal jugular veins” [10]. High central venous pressure can result in “increased episcleral venous pressure, leading to elevated IOP due to choroidal congestion and decreased aqueous humor outflow” [1]. In the literature, four out of the five cases of POVL after shoulder surgery involved patients in the BC position [1,4,6,7], with one case in supine positioning [5]. In our cases, each patient was placed in the BC position, and they all developed sudden-onset, painless vision loss in the postoperative period, with a rather surprisingly significant recovery of central VA in each affected eye.

In shoulder surgery, a retinal and posterior ciliary artery occlusion can occur as a result of ocular compression by a face mask or an improperly positioned headrest [6]. The compression may lead to an extreme increase in IOP, and subsequent optic nerve ischemia or CRAO [1]. Su et al. explain that “direct eye compression can cause venous congestion, with consequently elevated orbital pressure, which ultimately compromises circulation to ocular structures” [8]. Interestingly, Fournier and Velis explain that moderate retinal ischemia may present with lesser permanent damage, which can be identified by specific diagnostic ERG abnormalities [6]. In two of our patients (Cases 1 and 2), decreased ERG amplitudes, in accordance with retinal and mixed retinal and optic nerve ischemia, were found, respectively.

**Figure 17:**
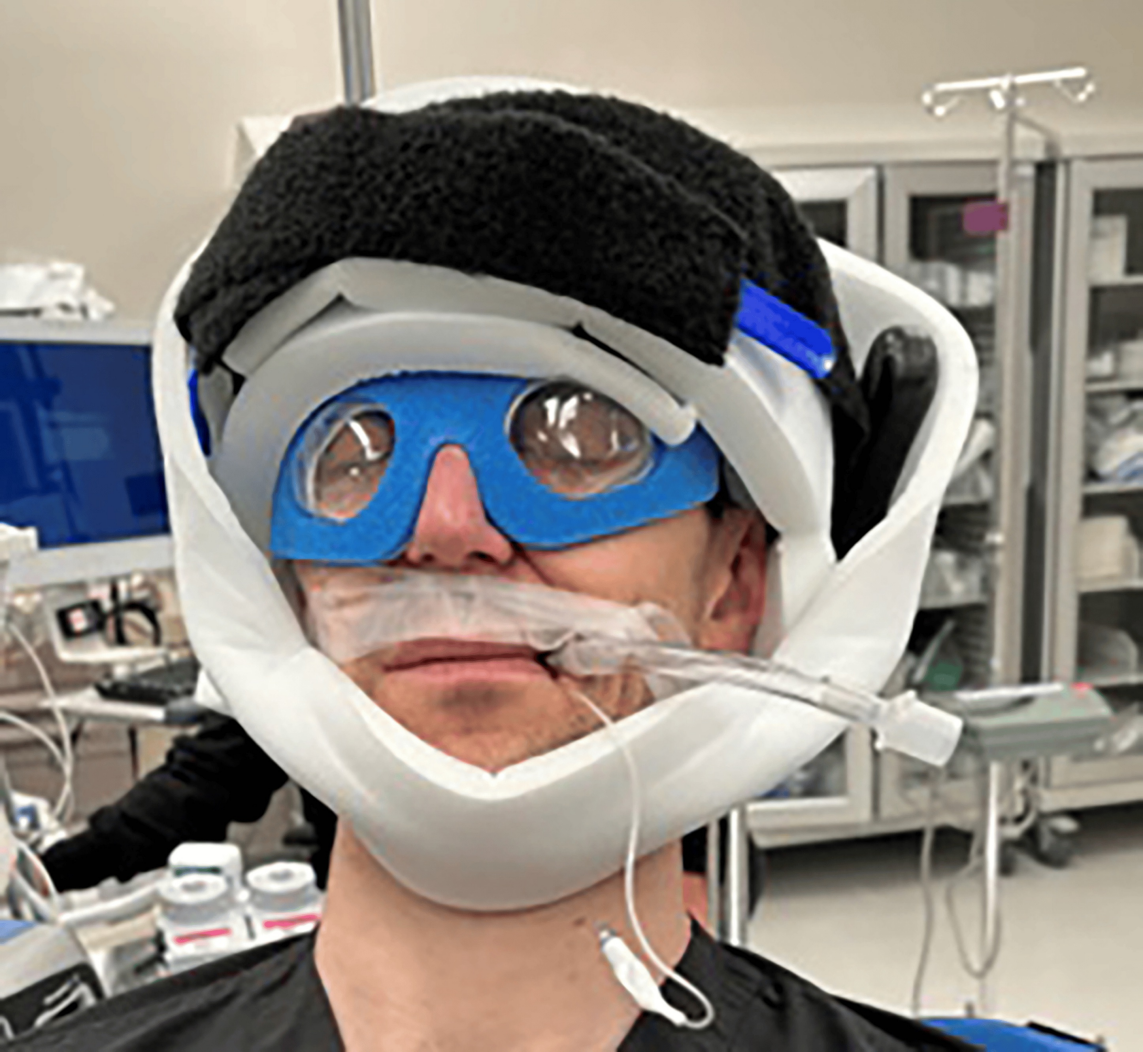
External photograph of beach-chair positioning with soft foam eye protection - front view This figure is a representational image (clinicians providing a demonstration).

**Figure 18:**
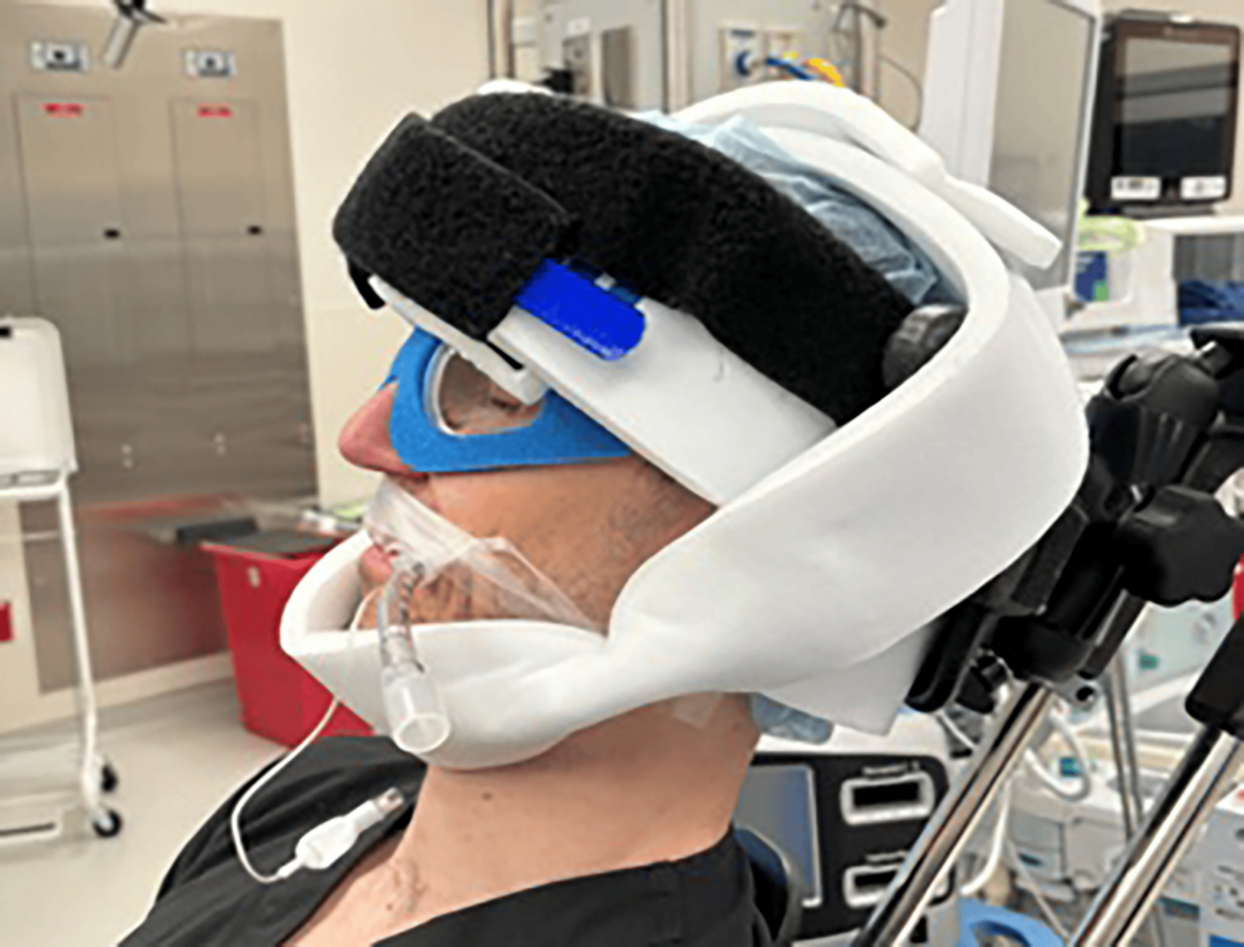
External photograph of beach-chair positioning with soft foam eye protection - side view This figure is a representational image (clinicians providing a demonstration).

Most orthopedic surgeons use eye goggles to protect the eyes during shoulder surgery, regardless of the LD or BC positions. The tight nature of the goggles surrounding the orbit might lead to inadvertent pressure on the globe with slight changes in patient position. Mumith and Scadden recommend adequate, but not excessive, padding of the eyes to prevent unnecessary extraocular pressure [7]. The use of cerebral oximetry in patients during shoulder arthroscopy in the BC position has also been recommended to aid in the recognition and management of decreased cerebral perfusion during surgery [10]. In addition, high-risk patients with expected prolonged operative time and/or substantial blood loss should be positioned so the head lies at or above the level of the heart in a neutral forward position, and intraoperative eye swelling and pressure should be checked consistently [3]. We propose the use of soft foam eye protection, as used in retina surgery, and a relaxed yet secure padded head strap to decrease the risk of POVL (Figures 17-18). The foam eye protection would provide intraoperative eye safety, as well as decrease the risk of globe compression during the procedure.

## Conclusions

POVL after shoulder surgery is a serious condition, with a risk of permanent vision loss. Ocular manifestations of POVL are variable and can include retinal and optic nerve ischemia. The etiology of POVL after shoulder surgery is multifactorial, owing to variations in operative positioning, external compressive forces on the orbit, and individual patient risk factors. Therefore, it is important to minimize risks and optimize both patient positioning and eye protection to promote patient safety and favorable outcomes.
